# 

**Published:** 1886-04-20

**Authors:** 


VOL. XVI. APRIL 20, 1886. NO. 4.

### THE SOUTHERN MEDICAL RECORD.

EDITORS:

T. S. POWELL, M. D. R. C. WORD, M. D.

COLLABORATORS:

J. McF. GASTON, M. D.,
W. D. BIZZELL, M. D.,
W. P. NICOLSON, M. D.,
E. VAN GOIDTSNOVEN, M. D.,
G. G. ROY, M. D.,
A. G. HOBBS, M. D.,
W. S. ELKIN, M. D.,
W. A. CROW, M. D.

**Terms: $2.00 per Annum, in Advance; 4 Copies $6.00; Single Copies 20 Cents.**

Address R. C. WORD, M.D., Managing Editor, Atlanta, Ga.

Published and Entered as Second-class Matter at ATLANTA, GA.

